# Isolation and identification of metallotolerant bacteria with a potential biotechnological application

**DOI:** 10.1038/s41598-024-54090-0

**Published:** 2024-02-13

**Authors:** Jonathan Parades-Aguilar, Kadiya Calderon, Sarai Agustin-Salazar, Pierfrancesco Cerruti, Veronica Ambrogi, Nohemi Gamez-Meza, Luis Angel Medina-Juarez

**Affiliations:** 1 Departamento de Investigaciones Científicas y Tecnológicas, Universidad de Sonora, Blvd. Luis Donaldo Colosio s/n, Entre Reforma y Sahuaripa, Edificio 7G, Col. Centro, C.P. 83000 Hermosillo, Sonora Mexico; 2 Institute for Polymers, Composites and Biomaterials (IPCB-CNR), Via Campi Flegrei 34, 80078 Pozzuoli, NA Italy; 3 Department of Chemical, Materials and Production Engineering (DICMAPI), University of Naples Federico II, Piazzale Tecchio 80, 80125 Naples, Italy

**Keywords:** Biotechnology, Microbiology, Environmental sciences

## Abstract

Mining has led to severe environmental pollution in countries with exhaustive mining production and inadequate industrial waste regulation. Microorganisms in contaminated sites, like mine tailings, have adapted to high concentrations of heavy metals, developing the capacity of reducing or removing them from these environments. Therefore, it is essential to thoroughly characterize bacteria present in these sites to find different ways of bioremediation. In this regard, in this study, an enrichment and isolation procedure were performed to isolate bacteria with lower nutritional requirements and high tolerance to Cu(II) and Fe(II) from two Sonoran River basin mining tails. Two *Staphylococcus* species and a *Microbacterium ginsengisoli* strain were isolated and identified from the San Felipe de Jesús mining tail. Also, three strains were isolated from the Nacozari de García mining tail: *Burkholderia cenocepacia*, *Sphingomonas* sp*.* and *Staphylococcus warneri.* Significant microbiological differences were found between the two sites. All these species exhibited tolerance up to 300 mg/L for Cu (II)–Fe (II) solutions, indicating their capacity to grow in these conditions. Moreover, a consortium of isolated bacteria was immobilized in two different biocomposites and the biocomposite with larger pore size achieved greater bacterial immobilization showcasing the potential of these bacteria in biotechnological applications.

## Introduction

In the northwest of Mexico, particularly on the border between Arizona and Sonora states, mining activity is one of the priority industrial sectors. This is due to the geological and climatic conditions that allowed the formation of mineral deposits and their exploitation. Sonora is considered the leading state in mining and metallurgical production in Mexico, owing to its substantial deposits of metallic minerals such as copper, gold and molybdenum, which are currently in operation. Additionally, it is also an important producer of non-metallic minerals such as graphite and wollastonite. The Sonoran River and the Yaqui River basins have played a prominent role in mining activities, hosting the main mining companies in the state. As a result of the large mining production, significant contamination is also generated^1^.

Since mines are generally located in municipalities near to watercourses, the incorporation of mining contaminants remains a risk factor. This can occur through the leaching of contaminants from mine veins using solvents or the mobilization of these wastes through wind or water dispersion. There is also a risk of landslides during rainfall events when tailings accumulate in piles^2^. Common contaminants resulting from mining activities include heavy metals (HMs). The ecological impact of HMs is mainly due to their high toxicity and the fact that they cannot be degraded. Exposure to high concentrations of metals can cause damage to human health, manifested at the molecular level through toxicity mechanisms derived from their capacity as cations and their high affinity for binding to other elements. This includes the blocking of functional groups in biomolecules such as proteins, displacement of cationic centers in crucial enzymes, and the generation of reactive oxygen species. This results in irreversible damage to proteins, lipids, and nucleic acids^3^. One of the main drawbacks of these pollutants is their ability to resist degradation and their bioaccumulation, causing damage to the liver, nervous system, reproductive system, and carcinogenesis^4^. In the specific case of Sonora, Mexico the notable presence of copper, Cu (II), and iron, Fe(II), in sediments and water bodies is highlighted^5,6^. These two metals are found in higher quantities and can have detrimental effects on the environment and human health. Cu (II), for instance, can impact aquatic life and induce toxicity in sensitive organisms. On the other hand, excessive Fe(II) can contribute to the eutrophication of water bodies, negatively affecting water quality and local biodiversity. High concentrations of iron in drinking water have been associated also with potential health concerns, such as gastrointestinal issues^7,8^.

In this regard, efficient and sustainable ways to remove metals from the environment are priority^9,10^. A possible way to achieve metal bioremediation is using microorganisms, such as bacteria present in the contaminated sites, which are able to adapt to high concentrations of metals, and to develop mechanisms for removing HMs from the environment^11–14^. Therefore, isolation of bacteria from sites with high metal concentration is a key step to better understand the bioremediation processes and exploit them for biotechnological purposes. Until now, little is known about the diversity of bacteria in mining tails in Sonora. The aim of the present work was to isolate and identify a group of bacteria with lower nutritional requirements and high tolerance to Cu(II) and Fe(II) from two mining tails from the Sonoran River basin in Mexico, in order to be applied in a promising bioremediation system.

## Results

### Samples characterization

Soil samples obtained from each mining tail presented differences in metal concentration and pH (Table 1). San Felipe de Jesús had a more acid pH, and higher concentration of all the analyzed metals. The latter could allow the presence of bacteria more tolerant to high concentrations of metals. The higher concentration of Pb and Zn in SFJ mine tailings can also be attributed to the waste's original composition. Similarly, this phenomenon occurs with Nacozari de García (NG), where despite having lower metal concentrations, the Cu concentration is most akin to SFJ, and it is also due to the waste's original constitution.

**Table 1:** pH values and concentration of total metals (mg/kg) of mine tailings samples.

Mine tailing	pH	Cu	Fe	Pb	Cr	Cd	Zn
NG	3.63 ± 0.01	269.4 ± 10.9	18,640 ± 1239	1661.2 ± 79.8	3.5 ± 0.3	2.4 ± 0.2	47.7 ± 3.2
SFJ	2.49 ± 0.02	376.3 ± 4.5	139,364.9 ± 5046	8981.63 ± 79.2	12.3 ± 0.6	53.7 ± 0.2	6049 ± 55.1

Mean ± standard deviation of three replicates. *SFJ: San Felipe de Jesús mine tailing, NG: Nacozari de García mine tailing.

### Enrichment and isolation of bacteria

**Figure 1:**
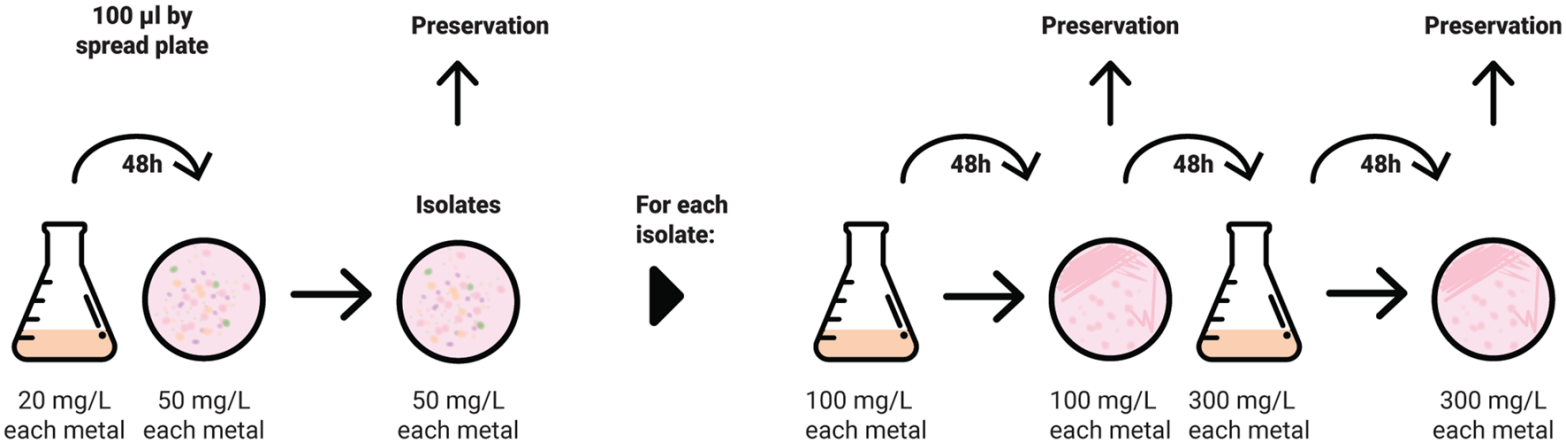
Isolation process of strains performed after enrichment process.

**Figure 2:**
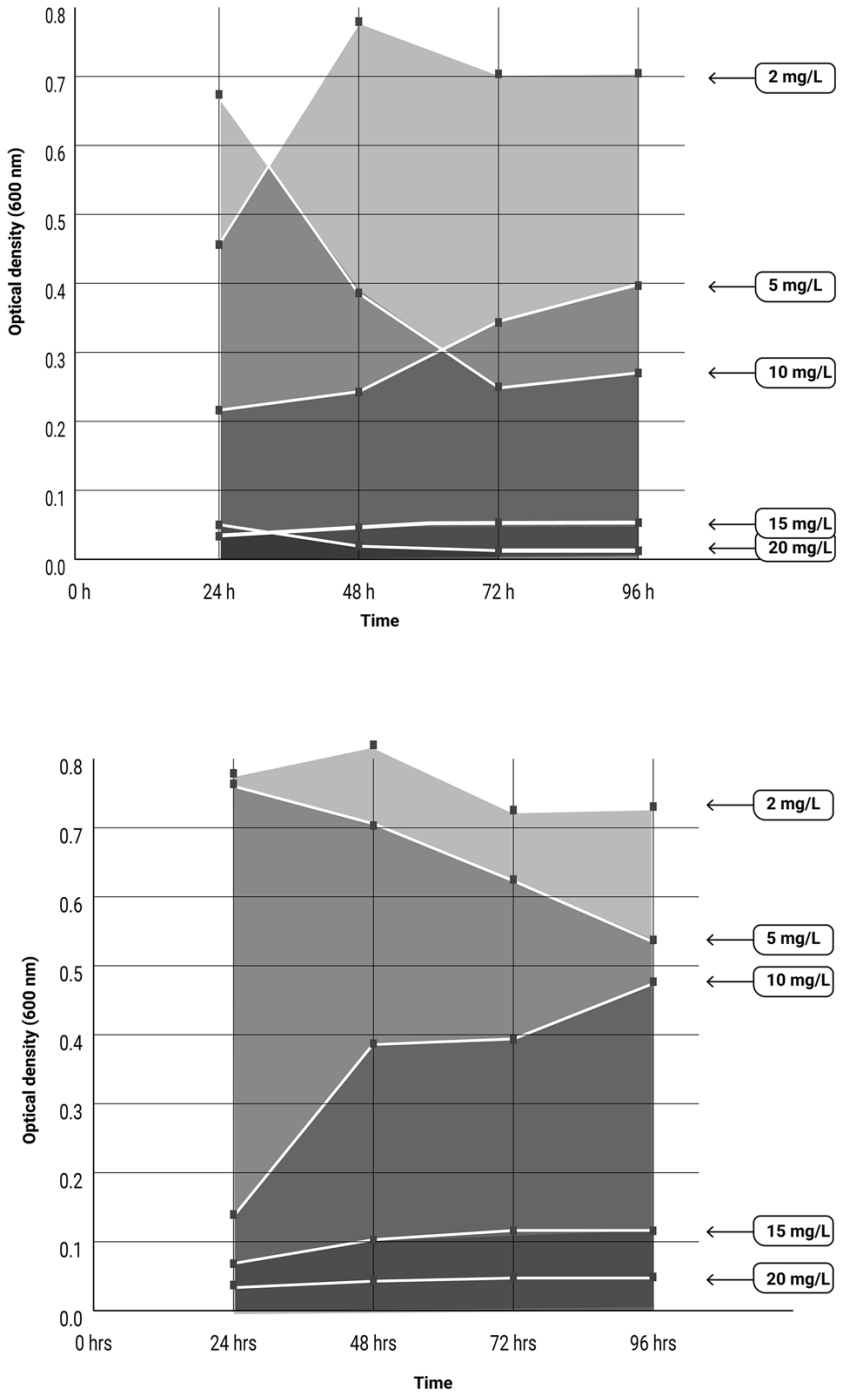
Growth curve (optical density, 600 nm) of the bacteria from (**A**) Nacozari de García and (**B**) San Felipe de Jesús samples during the acclimatation process with increasing concentrations of metals (Cu and Fe).

The bacteria isolation and the enrichment process were performed as reported by Majumder et al.^15^ with some modifications, as in this case bacteria were obtained from mining tails (Fig. 1). Bacteria with low nutritional requirements and tolerant to ascendant concentrations of Cu(II) and Fe(II) were isolated and analyzed from the consortium of each mining tails by optical density determination through time (Fig. 2). The isolated bacteria exhibited reduced growth with ascendant concentration of metals. Notably in NG bacteria, this trend was particularly pronounced in the concentration range between 10 and 20 mg/L, while from 15 to 20 mg/L the bacteria remained stable. In contrast, for the SFJ bacteria, bacterial proliferation was high at 10 mg/L, with a gradual increase in growth observed as the metal concentrations increased.

Once the acclimated consortium of each mining tail grew in 20 mg/L, regrowth in peptone yeast extract agar was performed in plates with 50 mg/L of each metal to be isolated and then identified. Three metallotolerant strains were isolated from SFJ mining tail, and three for NG. The strain name ID, gram stain and phenotype results of each isolate are shown in Table 2. SFJ bacteria were predominated gram positive, while NG predominated gram-negative bacteria. Additionally, SFJ exhibited more cocci morphology, while NG had more bacilli. The lowest concentration of the combination of metals that inhibited the growth of each isolated microorganism was found to be 300 mg/L. This concentration coincided with the level of Cu present in each mining tail. All isolated bacteria were identified based on their similarity percentage of identity using GenBank sequences by BLASTN (Table 3). The GeneBank accession numbers for the 16S rRNA sequences are PP157099-PP157104.

**Table 2:** Characterization of isolated bacteria by sampling site.

Mine tailing	Strain name ID	Gram stain and bacteria form	Phenotype color	Image
SFJ	SFJ02	Gram-positive cocci	Light brown	
	SF008	Gram-positive bacilli	Yellow	
	SFJ09	Gram positive cocci	Light-colored	
NG	NG01	Gram-negative bacilli	Light-colored	
	NG02	Gram-positive cocci	Light brown	
	NG06	Gram-negative bacilli	Yellow	

*SFJ: San Felipe de Jesús mine tailing, NG: Nacozari de García mine tailing.

**Table 3:** Molecular characterization of isolated bacteria by sampling site.

Mine tailing	Strain name ID	Phylum	Family	Closest relative (based on 16S rRNA gene)	Similarity percent	Overlap (nucleotides)	Accession number of closest hit
SFJ	SFJ02	*Bacillota*	*Staphylococcaceae*	*Staphylococcus epidermidis*	99.48%	1157	MT585400.1
	SFJ08	*Actinomycetota*	*Microbacteriaceae*	*Microbacterium ginsengisoli*	99.07%	1180	GQ181053.1
	SFJ09	*Bacillota*	*Staphylococcaceae*	*Staphylococcus pasteuri*	99.47%	1128	MG996881.1
NG	NG01	*Pseudomonadota*	*Burkholderiaceae*	*Burkholderia cenocepacia*	99.88%	1474	CP054820.1
	NG02	*Bacillota*	*Staphylococcaceae*	*Staphylococcus warneri*	99.15%	1164	OP269706.1
	NG06	*Pseudomonadota*	*Sphingomonadaceae*	*Sphingomonas* sp.	99.47%	1132	JQ660208.1

*SFJ: San Felipe de Jesús, NG: Nacozari de García.

### Immobilization of bacteria

**Figure 3:**
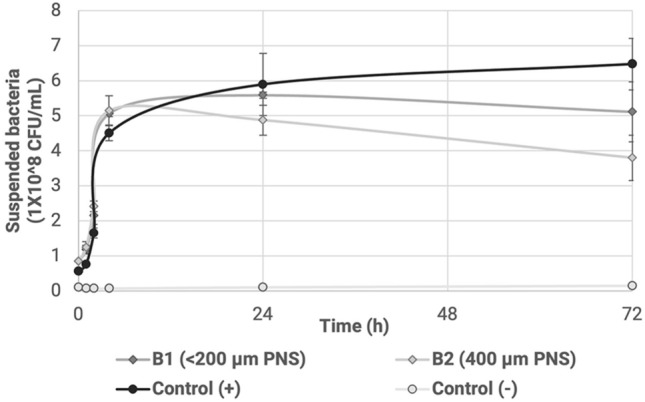
Immobilization of bacteria over time with two biocomposites.

A difference of almost 2 × 10^8^ suspended cells were observed in the immobilization tests between the control used and the biocomposites over an extended incubation period lasting up to 72 h, where a lower concentration of suspended bacteria compared to the control indicates a higher bacteria immobilization within the material. Although there is no statistically significant difference in the immobilization tests performed between both materials, the biocomposite with a larger pecan nutshell particle size, which was B2, showed a stronger biofilm and better immobilization (Fig. 3), which was subsequently confirmed through SEM of samples cultivated in an extended incubation period of two months. In the case of B1, there was no visible proliferation in the SEM images even from the beginning of the experiment.

**Figure 4:**
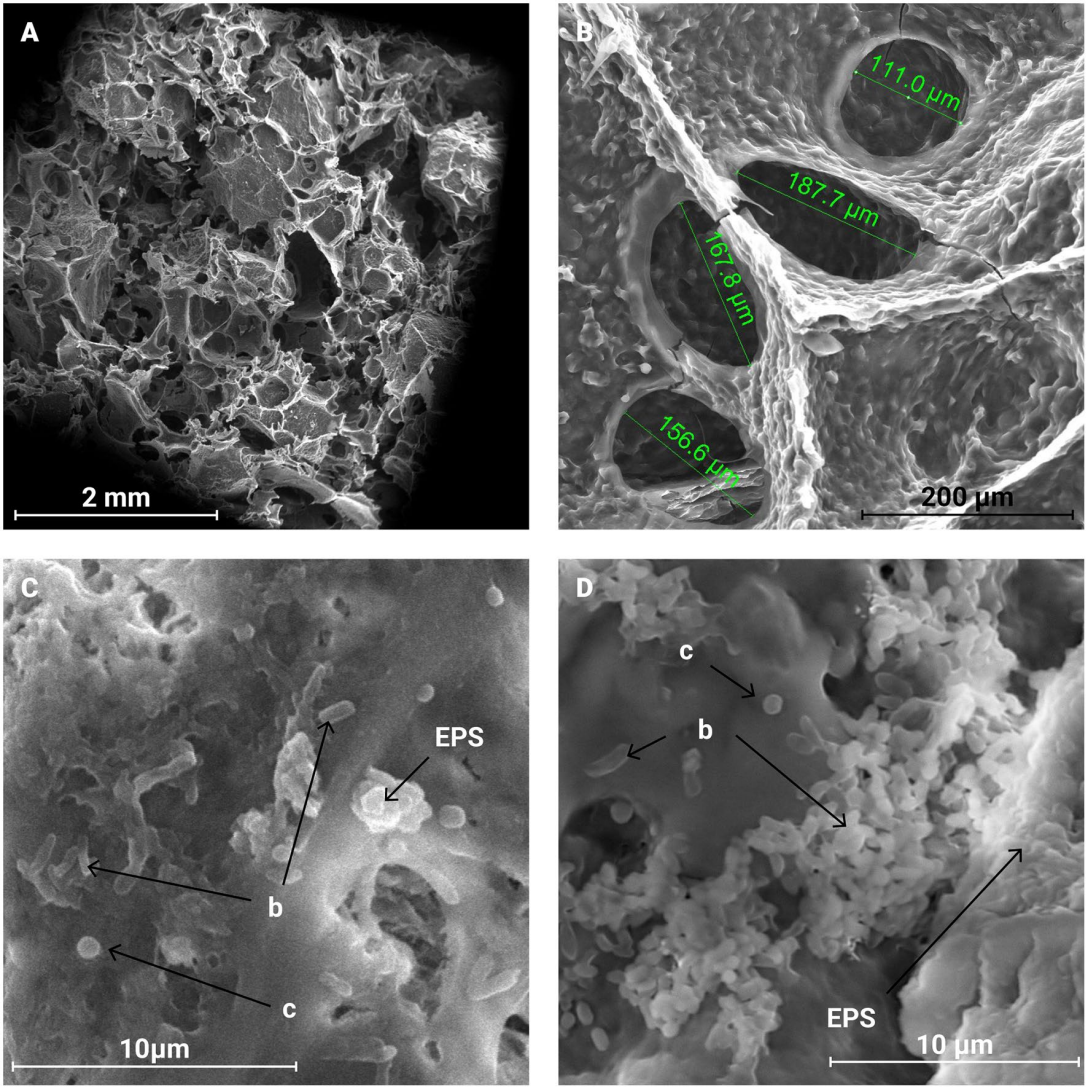
Immobilization of the isolated consortium in the 400 μm pecan nutshell biocomposite. (**A**) Biocomposite structure. (**B**) Biocomposite pore size. Bacteria immobilized in the 400-mesh biocomposite after an incubation period of (**C**) 1.5 months and (**D**) 2 months. * Letter b within the image indicates the presence of bacillary shapes, letter c indicates the presence of coccoid shapes while EPS refers to extracellular polymeric substances.

The SEM micrographs showed that B2 is a heterogeneous and porous material (Fig. 4A and B). The bacteria immobilized within the material correspond to the structure of the bacteria comprising the consortium (bacilli and cocci), with a major predominance of bacilli, which could match to the isolated *Burkholderia cenocepacia and Sphingomonas* sp., with an average size of 1–2 µm (Fig. 4C and D). They formed aggregates and secreted extracellular polymeric substances on the material’s pores that suggest that the biocomposite is a good support for the isolated bacteria.

## Discussion

Our study exhibits that the conditions in both mining tails facilitated the presence of bacteria with tolerance to acidic environments and elevated concentrations of metals, with a particular emphasis on the conditions found in SFJ, which exhibit higher concentrations of metals and greater acidity. The elevated concentrations of metals and acidity in the mine tailing may be linked to the site’s age. Importantly, the predominant use of selective flotation of mineral extraction in these mine tailing could also significantly impact acidity. Additionally, it is crucial to recognize that acid mine drainages can originate in mines due to the oxidation of sulfate-bearing materials^16^.

Chemical composition has been previously characterized for both mine tailings. For SFJ, results were in the range obtained by Del Rio-Salas et al.^17^ for Cd (98–665 mg/L), Cu (338–5691 mg/L), and Pb (832–14,162 mg/L). These authors also analyzed As and Sb, obtaining results between 1306–16,756 and 82–492 mg/L, respectively. In the case of NG, results were comparable to the concentrations range of mine tailings on the outskirts of the municipality of NG analyzed by Peña-Ortega et al.^18^ for Cu (216.2–1194.5 mg/L) and Zn (49.7–394.7). In comparison with previous reports for this mine tailing, it seems that Pb concentration has increased.

Bacteria from SFJ likely avoided the toxic effects of the metals used, as they were present in sediments on a site with higher concentrations of metals. The resistance of tolerant bacteria to metals can be explained by the presence of specific genes in their genome that encode proteins such as metallothioneins^19^. These kinds of proteins enable the bacteria to withstand toxic effects or even utilize metals like Cu(I) as electron acceptor, acting as a source of energy^13,20^. Additionally, examples of bacterial resistance to heavy metals may involve features such as cell impermeability facilitated by the presence of exopolysaccharides that adsorb metals^21^. The resilience of bacteria to metal toxicity has been previously reported by Escamilla-Rodrigez et al.^22^ who observed an increase in bacterial concentration (CFU) in response to Cu and Hg. Bacteria tolerance to Cu and Fe can also be explained by the use of these metals as micronutrients for different metabolic pathways, or the facility of elements such as Cu for the formation of complexes with organic matter and its adsorption onto particulates (Fe–Mn-oxyhydroxides) such in the case of some *Bacillus* species^23^. Furthermore, the isolated bacteria may exhibit tolerance to Pb, a metal that is prominently present in both mine tailing, indicating a potential biotechnological use of the isolated species. Pb bioaccumulation in bacteria has been reported previously by Tiquia-Arashiro et al.^24^.

Common taxa in environments like mine tailings or even in rhizosphere environments include microorganisms from phyla *Actinomycetota, Pseudomonadota*, and *Bacteroidota*. The differences of bacteria groups could be associated to the abiotic factors in each site favoriting the development and prevalence of specific groups, Predominance of *Actinobacteria* has been observed in arid soils^25^. In the present study more bacteria from *Bacillota* and *Actinomycetota* phyla were identified. Three strains tolerant to metals were identified in the SFJ mining tail: *Staphylococcus epidermidis, Staphylococcus pasteuri and Microbacterium ginsengisoli* and three in NG: *Burkholderia cenocepacia*, *Sphingomonas* sp*.* and *Staphylococcus warneri.*

Escamilla-Rodriguez et al.^22^ also reported about three *Staphylococcus* species isolated from water of a mining area in Zacatecas, Mexico. Two of these bacteria had one hundred percent of growth when they were incubated in plates supplemented with Cu, in comparison with the initial population. They also had high growth rate in other metals tested (100 and 167% for Cr, 120% for Zn, 140% for Ag, 66 and 120% for Hg and 20 and 66.7% for Co, respectively). As indicated by Gadd^26^, the primary binding sites for metal cations in the cell walls of Gram-positive bacteria are the carboxyl groups within peptidoglycan. Furthermore, it has been reported that *S. epidermidis* has developed the ability to tolerate and grow in the presence of moderate concentrations of Cu. Resistance to Cu is partly attributed to the presence of specific genes in its genome encoding copper transport proteins and copper resistance proteins. These proteins enable the bacterium to withstand the toxic effects of Cu(I) and utilize such as a micronutrient or cofactor in key enzymes of important biological processes^27^. Such mechanisms suggest the adaptability of *S. epidermidis* to environmental copper stress, potentially explaining the presence of various *Staphylococcus* species in both locations in the present study, particularly due to the high copper concentrations at both sites.

In a similar way, Toribio-Jimenez et al.^28^, and Toledo-Hernández et al.^29^ isolated strains of *Staphylococcus* sp. from the rhizosphere of the mining tail El Fraile, located in Guerrero, Mexico, and in this case evaluated their capacity to bioaccumulate Ag(II). In the case of *S. pasteuri* isolated from Iranian mine calcareous soils has shown to remove 98.71% of Pb, 97.15% of Cd, and 94.83% of Zn by bioprecipitation due to their capacity to produce urea^30^, highlighting the potential biotechnological application of the *Staphylococcus* species isolated in the present work for metal recovery by different mechanisms.

On the other hand, regarding the *Microbacterium* sp isolated in the present work, a study by Jroundi et al.^23^, reported that *Microbacterium* genus accounted for approximately 1.03% of the relative abundance in deep-sea sediments from the westernmost Mediterranean. Furthermore, the study revealed a positive correlation between the presence of metals, such as Cu, Co, Zn, Pb, within this genus, as well as with other genera, (including *Bacillus*, *Bacillales*, *Paenisporosarcina*, *Megasphaera*, and the *Enterobacteriaceae* family). The authors suggested that this correlation may be attributed to changes in oxygenation induced by the presence of metals from anthropogenic activities, which may promote the proliferation of these bacteria. Similar conditions to those found in mining tailings such as those found in San Felipe de Jesús in the present study. *Microbacterium* as well as the strain of *Sphingomonas* isolated in the present work were also capable to develop pigments, offering another potential application for these bacteria.

In the case of *Sphingomonas*, it is an endophytic bacterium that has been previously isolated from *Sedum alfredii* roots and promote zinc extraction, so it could be used to enhance processes of phytoremediation^31^. *Sphingomonas paucimobilis* has been also used to degrade different organic pollutants, but in addition it has been proved that it serves as an adsorbent for the removal of Cu(II) ions from industrial effluents^32–34^. Furthermore, it has been reported that some strains of *Sphingomonas* and *Microbacterium* can produce yellow pigments as a result of their carotenoid synthesis metabolism, such as zeaxanthin and nostoxanthin^35^. These carotenoids have potential antioxidant properties, which can protect cells against oxidative stress, possibly explaining the tolerance of this strain to heavy metals, and even to gamma-ray irradiation, as reported by Asker et al.^35^ for a species of *Sphingomonas* isolated from freshwater samples collected at Misasa (Tottori, Japan), a region known for its high natural radioactivity, and designated as *S. jaspsi*. Furthermore, high concentrations of heavy metals can lead to the generation of free radicals in the environment, and carotenoids produced by these strains may neutralize them, preventing the harmful effects of oxidation. Therefore, the carotenoids of these species can be harnessed for their multifunctional roles, including applications in various industries such as food, pharmaceutical, cosmetic, and textile^36^. At the same time, these compounds make these species useful in bioremediation due to their antioxidant activity, allowing for increased bacterial tolerance and enabling subsequent remediation mechanisms like biosorption or bioprecipitation^23,30^.

In the case of the *Burkholderia* genus*,* it is a plant-associated genus that has previously been isolated from the rhizospheric soils in the same mining tail of Nacozari de García^25^. The genus *Burkholderia* is known for its capacity to remove metals. Jiang et al.^37^ isolated and identified a metal-resistant strain named *Burkholderia* sp. J62 from metal-contaminated soils, demonstrating its ability to solubilize metals such as Pb and Cd. Similarly, Yang et al.^38^ found that a *Burkholderia* sp. strain designated as Z-90 exhibited a removal efficiency of 44% for Zn, 32.5% for lead, 52.2% for Mn, 37.7% for Cd, 24.1% for Cu, and 31.6% for As. It was also observed that the removal capacity of these metals was attributed to mineral adhesion to the strain and the formation of a metallic complex with a glycolipid biosurfactant excreted extracellularly. The capacity to produce these extracellular polymeric substances makes bacteria potentially suitable for immobilization and application in bioremediation systems designed for metal removal. Similarly, *Staphylococcus epidermidis* strain that producing brown pigment, as observed in the present work, exhibit a greater capacity to form biofilms, as reported by Yao et al.^39^. Therefore, the isolation of bacteria with this capability enables their immobilization, a process that was subsequently analyzed and confirmed in the present work by SEM^40,41^.

The increased interest in the application of immobilized microorganisms in bioremediation processes arises from the higher biodegradation efficiency observed when compared to free microorganisms^14,42–45^. Additionally, as presented by Rahman et al.^46^, immobilized bacterial cells could be recovered and reused, lowering the cost of a possible remediation process, and when using a consortium or community of bacteria instead of individual strains in a biotechnological application, the removal rate increases^42,47^. In the present study, the selection of pecan nutshell biocomposite materials was motivated by their promising attributes, including renewability, biodegradability, and functionality, making them advantageous for various applications such as bioremediation, wastewater treatment, and sustainable biofilters^48,49^.

The proliferation of bacteria in the biocomposite with larger pecan nutshell particle size (B2) can be explained by the larger pore diameter in such material, which ranges between 100 and 200 µm (Fig. 4B). These pores can serve as accumulation channels, as they increase the surface area^50,51^ and, at the same time, can facilitate the transfer of ions for the removal of impurities or contaminants^52^. Darmayati et al.^53^ performed a study to analyze the immobilization of different bacterial strains on porous rock carriers after being stored at 25 °C for three months, conditions similar to those used in the current work. They attribute the differences in cell immobilization observed among the materials to variations in physical characteristics such as porosity and surface area, because materials like zeolites, which have a rough surface, tend to increase the surface area, leading to greater cell immobilization^42,54^.

It is also noteworthy to mention the presence of extracellular polymeric substances (biofilms) secreted by the bacteria upon adhering to the biocomposite. The formation of biofilms by bacteria could be a response to stressful environmental conditions and serves as a protective mechanism for bacteria. Additionally, it can indicate the tolerance of the isolated bacteria to adverse conditions, such as nutrient scarcity^55^. This tolerance may be attributed to the isolated bacteria’s capacity to thrive under minimal nutrient conditions.

Extended periods of time exceeding one month as in the present study, also promote the optimal growth of microorganisms, as demonstrated by Sarioglu et al.^56^. Their one-month immobilization of *Morganella morganii* STB5 on the surfaces of electrospun polystyrene and polysulfone webs resulted in a strong attachment of bacterial cells and this timeframe was considered adequate for conducting further studies on Cr (VI) removal.

In the case of immobilization on organic materials, Lupascu et al.^57^ presents the adsorption of *Bacillus subtilis* and *Bacillus cereus* on activated carbon derived from apricot kernels. In this case, immobilization occurs only on the exterior of the material since the pores generated are smaller than the size of the bacteria. Furthermore, they identify that bacteria are immobilized through interactions between basic groups from their structure (such as amines, amides, and pyrenes) with carboxylic, lactonic, and phenolic groups on the surface of the activated carbons. Similar interactions may be occurring between the immobilized bacteria in the present study and the pecan nutshell biocomposite, as well as in any organic material, especially those of plant origin with similar functional groups.

Despite this, few studies have focused on immobilizing bacteria in agro-industrial wastes. Gallardo-Rodríguez et al.^14^ utilized *Furcraea andina* fibers to immobilize *Pseudomonas* bacteria, employing them as a biofilter for lead removal. Their findings demonstrate the effective use of natural fibers from this plant for both *Pseudomonas* immobilization and metal removal. On the other hand, yeast cells such as *Saccharomyces cerevisiae* have been immobilized on *Raphia farinifera* fibers obtaining a high adsorption capacity for the removal of Pb (II). Therefore, the present study is one of the few that elucidates the potential application of all the isolated bacteria, immobilized within an organic material to enhance their bioremediation capabilities.

In conclusion, the present study demonstrated the disparity in the cultivated bacterial diversity found between two mining tailings within the Sonoran River basin. It identifies species that had not been previously reported from each site and demonstrates their tolerance to Cu (II) and Fe (II) solutions of up to 300 mg/L. The isolation process selectively yielded bacteria with high tolerance to Cu and Fe tolerance as well as those with low nutritional requirements. Among the isolated strains, *Staphylococcus epidermidis*, *Staphylococcus pasteuri*, and *Microbacterium ginsengisoli* from SFJ mine tailing, along with *Staphylococcus warneri*, *Burkholderia cenocepacia*, and *Sphingomonas* sp. from NG mine tailings, exhibit promising biotechnological potential for metal removal applications as previously presented in the literature. Furthermore, bacteria like *Microbacterium* and *Sphingomonas*, known for their pigment production capability, not only add value to these isolated bacteria for various applications but also have the potential to enhance their bioremediation activities. Moreover, the study successfully demonstrates the immobilization of these isolated bacteria through biofilms within an organic natural-origin biocomposite, making them ideal candidates for inoculating remediation systems aimed to heavy metal removal. Additionally, this work showcases the potential use of an agro-industrial waste product like pecan nutshell, which represents a cost effective and biodegradable product for possible remediation systems in which these bacteria can be applied. Hardest efforts have been performed in order to advance in these fields nevertheless, further research is needed to elucidate their mechanisms to decontaminate polluted environments.

## Methods

### Sampling sites

Mine tailings soil samples were collected during March 2021 from two abandoned sites located in two different historic mining areas in Sonora, Mexico: San Felipe de Jesus (SFJ) and Nacozari de García (NG) mine tailings. SFJ mine tailing is located in the San Felipe de Jesús municipality (N 29° 55′ 15″–29° 49′ 18″ and W 110° 260 12″–110° 10′ 02″), which is a semi-arid land with a mean temperature of 25.3 °C during July and August, the months with the highest temperatures, and an annual average precipitation of 468.8 mm. This tailing deposit has an estimated area of around 16,300 m^2^ and an approximate volume of 209,455 tons of mining wastes. Since 1900, the metallic recovery of Pb, Zn, Cu, Ag was carried out by selective flotation^16^. Additionally, it's important to note that the mine tailing is located at less than one kilometer from the town, which can potentially impact the population, particularly the existing agricultural sites and the Sonora River, which flows right alongside the town.

The second sampling site is located in Nacozari de García municipality (N 30° 21′ 24″–30° 21′ 29″ and W 109° 40′ 57″–109° 40′ 55″). This place has a predominant temperate semi-dry climate area with an average temperature of 35 °C during July, month with the higher temperature, and an average annual precipitation of 490 mm. Metal exploitation, mainly Cu, began in 1900 and continues to the present day, with the current estimated volume of around three million tons of mining wastes^16,58^. In this case, the mining site is located within the city, which increases the potential risks for the local population due to its proximity to mining waste.

The superficial sampling of sediments was performed according to the method described in NMX-AA-132-SCFI-2016^2,59^. Five samples from each mining tail, with a minimum distance of 20 m, were collected at five randomly accessible sites in each mining area and pooled to obtain a composite sample of each mining tail. The samples were manually collected (30 cm) using a stainless-steel shovel and stored in sterile plastic bags until their immediately characterization. The collected mine tailing samples were completely homogenized, dried at 30 °C by 24 h and sieved through a 1 mm pore size to be used for physicochemical characterization and the isolation process.

### Physicochemical samples characterization

For the samples characterization, The pH was measured by a potentiometer method using the supernatant suspension of a mixture with each composite mine tailing sample: water ratio of 1:2. The total metal composition was determined by Atomic Absorption Spectrometry (AAS)^60,61^. Firstly, 0.5 g of soil composite from each mine tailing was totally digested with 0.5 mL HNO_3_, 0.5 mL HF and 0.5 mL HClO_4_ due the presence of silicates in the mining tailings^5^. A second reduction was performed using 2 mL of HNO_3_ and 2 mL HF followed by heating at 95 ± 5 °C of 2 mL HNO_3_ for 30 min. Finally, the sample was heated in 10% H_3_BO_3_ for 20 min at 95 ± 5 °C and filtered on Whatman paper filters No. 40. The samples were diluted in 2% HNO_3_ before being analyzed in a Perkin Elmer 3100 atomic absorption spectrometer.

### Enrichment and isolation of samples

For bacteria acclimatation process, 10 g of composite soil were exhaustively mixed with 100 mL of nutrient broth (0.5% peptone, 0.5% NaCl, 0.15% yeast extract, 0.15% meat extract) in 250 mL flasks per triplicate and with their respective blanks. The flasks were incubated at 30 °C and 150 rpm over 120 h. The supernatant from the flasks was then centrifuged in conical tubes (3200 g, 4 °C, 15 min), and the formed pellets were transferred to conical tubes. A process of enrichment of the bacterial culture with metals was performed as described by Majumder et al.^15^. This process consists in acclimation the obtained biomass to a mix of metals for six days. For this, 100 mL of minimal salt medium (MSM) (0.08% K_2_HPO_4_, 0.02% KH_2_PO_4_, 0.05% MgSO_4_, 0.005% CaSO_4_, 0.1% (NH_4_)_2_SO_4_, 0.001%FeSO_4_) was added to the pellets in the conical tubes with an initial concentration of glucose of 1 g/L (carbon source). The tubes were maintained in an incubator at 30 °C and 150 rpm during six days. Samples were centrifuged daily, discarding the supernatant, and progressively decreasing the concentration of glucose to reach 0 mg/L at the sixth day (1000, 800, 600, 400, 200 and 0 mg/L), and a concentration of metals of 20 mg/L Cu(II) and Fe(II) (0, 2, 5, 10, 15, and 20 mg/L) as pentahydrate copper sulfate and heptahydrate iron sulfate, respectively (Supplementary material Table 1). Each concentration was still stored at 30 °C and 150 rpm, and optical density determinations were performed after transitioning to the next concentration to determine the bacterial growth at each concentration.

After the enrichment, five tenfold dilutions on MSM of the bacterial culture with 20 mg/L of metals were prepared in MSM and 100 µl of each dilution was plated by using the spread-plate method in peptone yeast extract agar plates with 50 mg/L of each metal for the isolation. The plates were kept at 30 °C and analyzed every 24 h. When the isolated strains were obtained, the same process was repeated with 100 and 300 mg/L of each metal using plates with the same concentrations of metal^15^ as depicted in Fig. 1, to identify what was the maximum concentration that the bacteria can tolerate^62^. The isolates that grew up at each concentration of metals were preserved in culture broth and glycerol (50%) at − 80 °C.

### Identification of isolated bacteria

Gram stain^63^ and molecular analyses using 16S rRNA gene amplification and sequencing were performed in order to identify the isolated colonies using the isolated bacteria grown in nutrient broth in an incubator at 30 °C after 48 h. DNA of each isolate was extracted by Fast DNA Spin Kit for Soil (MP Biomedicals, Solon, OH, US) following the manufacturer instructions.

The total DNA quality was performed using a NanoDrop 1000 spectrophotometer (Thermo Fisher, Waltham, MA, US) using 1 μL of sample. For the further analyses, the samples with an absorbance ratio of 260/280 nm in the range > 1.8–2.0 were used. Total DNA integrity was analyzed by 0.8% agarose gel electrophoresis stained with ethidium bromide and visualized using Bio-Imaging System MiniBis pro photodocumenter (Bio America Inc., CA, US). The electrophoresis conditions were 50 V for 50 min in 1X TAE.

For PCR, universal primers 27F and 1525R were used for the amplification of the 16S rRNA gene^64^. HPLC-purified oligonucleotides were purchased from Sigma, and GoTaq Flexi DNA Polymerase (Promega, Madison, WI, USA) was used for all PCRs, which were performed in a Bio-Rad T100 Thermal cycler (Bio-Rad, Hercules, CA, USA). The PCR mixture was 10 μl of GoTaq FlexiBuffer 1X, 3 μl MgCl_2_ 1.5 mM, 2.5 μl DMSO 5%, 0.5 μl of each primer (0.8 μM), 1 μl dNTPs 0.2 μM, 0.2 μl of 2U/μl of Polymerase Taq. All reactions were performed using 4 μl of DNA in a reaction of 50 μl. The amplification conditions were a touchdown phase with an initial denaturation at 95 °C for 5 min; 10 cycles of denaturation at 94 °C for 30 s, an annealing from 65 to 55 °C for 30 s during the 10 cycles, and extension at 72 °C for 30 s, and then a second phase of 25 cycles with denaturation at 94 °C for 30 s, annealing at 55 °C for 45 s, extension at 72 °C for 2 min, and final extension at 72 °C for 5 min. DNA sequencing of the amplicons was performed at Macrogen (South Korea). The sequences obtained were first aligned in ClustalX^65^ and compared with GenBank sequences using BLAST^66^.

### Immobilization of bacteria

The capacity of the isolated bacteria to be immobilized on a solid matrix was evaluated using a consortium of the isolated bacteria from the stocks of 50 mg/L of each metal. The evaluation employed the same proportion of each isolated strain as an inoculum to form a consortium with a concentration of 8.4 × 10^8^ CFU/mL^14^. These tests were conducted using two biocomposites as immobilization materials, consisting of albumin-starch as the polymeric matrix, and an agro-industrial waste like pecan nutshell as the fiber filler. The two biocomposites varied as for the size of the pecan nutshell particles used, which were designated as B1 (< 200 µm of PNS particle size) and B2 (400 µm PNS particle size), with the main difference being greater roughness and pore size in B2.

For these tests, 0.5 g of each biocomposite was placed in tubes with 10 mL of sterile nutrient broth, which had 1 mL of the bacterial consortium, obtaining 8.4 × 10^7^ CFU/mL^14^. The positive control had the same concentration of bacteria in nutrient broth without any biocomposite. The analysis of immobilization results was performed by measuring the optical density (600 nm) of the suspended bacteria and by means of CFU based on McFarland Standards over a 72 h period. Analyses were performed in triplicate and were compared: (a) with a positive control with culture medium with the consortium of bacteria, (b) a control using each biocomposite without inoculated bacteria, and (c) a negative control composed only of culture medium.

Additionally, to confirm the immobilization of bacteria over an extended period within each biocomposite, the formation of biofilms after six and eight weeks, where the material was kept in an incubator at 30 °C with weekly changes of culture medium (70% each week), carefully avoiding any disturbance of the material were analyzed using scanning electron microscopy (SEM) with a Tabletop Microscope TM3030Plus, (HITACHI, Tokyo, Japan), and analyzed by the provided software (Environmental Science Laboratory, ERNO, UNAM).

For these analyses, the samples were initially fixed with 5% v/v glutaraldehyde (Sigma Aldrich, San Luis, MO, US) in 1 X phosphate buffer saline (PBS) for 48 h at 4 °C. Subsequently they were washed with PBS and postfixed with 1% OsO_4_ for 2 h at 4 °C. After another wash with PBS, the samples were dehydrated using a series of increasing acetone solutions (ranging from 30, 50, 70, 90 and 100% acetone) before being subjected to analysis^54^.

### Ethical approval

This article does not contain any studies with human participants or animals performed by any of the authors.

### Supplementary Information

Supplementary Information.

## Data Availability

The original contributions presented in this study are included in the article; further inquiries can be directed to the corresponding author.
