# Supplementary material for: Isolation and identification of metallotolerant bacteria with a potential biotechnological application
Source: Sci Rep. 2024 Feb 13;14:3663. doi: 10.1038/s41598-024-54090-0 (PMC10864330; doi:10.1038/s41598-024-54090-0)
Supplement: Supplementary file 1 — Supplementary Information. [file 41598_2024_54090_MOESM1_ESM.docx]

**Table 1. Recipe of MSM media and concentration of metals and D-Glucose over time used to perform the isolation process.**

| **Day** | **Metals concentration (mg/L)** | **D-Glucose concentration (mg/L)** |
| --- | --- | --- |
| 1 | 0 | 1000 |
| 2 | 2 | 800 |
| 3 | 5 | 600 |
| 4 | 10 | 400 |
| 5 | 15 | 200 |
| 6 | 20 | 0 |
